# Machine Learning Prediction of Treatment Outcome in Late-Life Depression

**DOI:** 10.3389/fpsyt.2021.738494

**Published:** 2021-10-20

**Authors:** Adrienne Grzenda, William Speier, Prabha Siddarth, Anurag Pant, Beatrix Krause-Sorio, Katherine Narr, Helen Lavretsky

**Affiliations:** ^1^Department of Psychiatry and Biobehavioral Science, David Geffen School of Medicine, University of California, Los Angeles, Los Angeles, CA, United States; ^2^Medical Imaging and Informatics Group, Department of Radiological Sciences, University of California, Los Angeles, Los Angeles, CA, United States; ^3^Jane and Terry Semel Institute for Neuroscience and Human Behavior, University of California, Los Angeles, Los Angeles, CA, United States; ^4^Department of Neurology, David Geffen School of Medicine, University of California, Los Angeles, Los Angeles, CA, United States

**Keywords:** machine learning, pharmacology, prediction model, computational modeling, late-life depression (LLD)

## Abstract

**Background:** Recent evidence suggests that integration of multi-modal data improves performance in machine learning prediction of depression treatment outcomes. Here, we compared the predictive performance of three machine learning classifiers using differing combinations of sociodemographic characteristics, baseline clinical self-reports, cognitive tests, and structural magnetic resonance imaging (MRI) features to predict treatment outcomes in late-life depression (LLD).

**Methods:** Data were combined from two clinical trials conducted with depressed adults aged 60 and older, including response to escitalopram (*N* = 32, NCT01902004) and Tai Chi (*N* = 35, NCT02460666). Remission was defined as a score of 6 or less on the 24-item Hamilton Rating Scale for Depression (HAMD) at the end of 24 weeks of treatment. Features subsets were constructed from baseline sociodemographic and clinical features, gray matter volumes (GMVs), or both. Three classification algorithms were compared: (1) Support Vector Machine-Radial Bias Function (SVMRBF), (2) Random Forest (RF), and (3) Logistic Regression (LR). A repeated 5-fold cross-validation approach with a wrapper-based feature selection method was used for model fitting. Model performance metrics included Area under the ROC Curve (AUC) and Matthews correlation coefficient (MCC). Cross-validated performance significance was tested by permutation analysis. Classifiers were compared by Cochran's Q and *post-hoc* pairwise comparisons using McNemar's Chi-Square test with Bonferroni correction.

**Results:** For the RF and SVMRBF algorithms, the combined feature set outperformed the clinical and GMV feature sets with a final cross-validated AUC of 0.83 ± 0.11 and 0.80 ± 0.11, respectively. Both classifiers passed permutation analysis. The LR algorithm performed best using GMV features alone (AUC 0.79 ± 0.14) but failed to pass permutation analysis using any feature set. Performance of the three classifiers differed significantly for all three features sets. Important predictive features of treatment response included anterior and posterior cingulate volumes, depression characteristics, and self-reported health-related quality scores.

**Conclusion:** This preliminary exploration into the use of ML and multi-modal data to identify predictors of general treatment response in LLD indicates that integration of clinical and structural MRI features significantly increases predictive capability. Identified features are among those previously implicated in geriatric depression, encouraging future work in this arena.

## Introduction

Late-life depression (LLD) is a common disorder among community elderly associated with poor quality of life, increased risk for cognitive decline, and increased mortality, including suicide (1–3). Medical comorbidities and polypharmacy increase the complexity of treatment selection due to drug-drug interactions and heightened risk of adverse events (4). Decreased efficacy of antidepressants is observed with increasing age, likely attributable to increased somatic illness burden, ischemic or neurodegenerative brain changes, and/or suboptimal dosing by prescribers (5).

LLD treatment selection is currently guided by patient preference and trial and error. The search for treatment-response biomarkers has generated a wealth of genomic and neuroimaging data, however no candidate markers have transcended into routine clinical practice. Structural magnetic resonance imaging (MRI) features are appealing due the non-invasiveness of acquisition and relatively low cost. In LLD compared to healthy controls, gray matter volume (GMV) reductions are frequently observed in the fronto–striatal–limbic regions (6–9). Differences in GMV often associate to differences in antidepressant treatment response (10–13).

Early and aggressive intervention in LLD is critical to mitigating its devastating consequences. Machine learning algorithms have significantly advanced diagnostic and prognostic modeling of structural MRI data in numerous psychiatric disorders (14). Predictions from unimodal data, however, have produced often mixed results when applied to new data with high accuracy sometimes limited to the most severe forms of illness (15). Models that integrate multiple data modalities (e.g., clinical, imaging, biological), have shown superiority in diagnostic classification tasks (16–20). Such models, however, require a higher degree of expertise than unimodal models, both in design and in interpretation of results, especially when using “small” data (<100 observations (19). In the current study, we hypothesized that a multi-modal feature set would better predict depressive remission in patients with LLD compared feature sets containing only clinical or GMV variables.

## Methods

### Data Sources

Data were derived from two completed clinical trials of treatment of LLD (NCT01902004; NCT02460666, Supplementary Table S1) (21, 22). NCT01902004 spanned from January 2013 to January 2019, while NCT02460666 spanned January 2016 to November 2020. Informed consent was obtained from all participants prior to engaging in any research procedures and all procedures were approved by the Institutional Review Board at UCLA. Both studies employed a similar study protocol. Exclusion criteria were: (1) history of any psychiatric disorder (except for stable comorbid anxiety or stable comorbid insomnia); (2) acute suicidal ideation or suicide attempt within the past year; (3) severe or acute unstable medical illness or neurological disorder; or (4) dementia. Both studies required a diagnosis of major depressive disorder as defined by Diagnostic and Statistical Manual (DSM)-IV-TR or DSM-5. For the current analysis, inclusion criteria were set at: (1) age ≥ 60 years; (2) normal cognitive functioning as defined by a Mine Mental Status Exam (MMSE) score of 24 or greater; and (3) at least mild-moderate depression at treatment initiation.

### Treatments and Clinical Assessments

For NCT01902004, participants were required to be free of antidepressant medication prior to enrollment, then randomized to receive either escitalopram/placebo or escitalopram/memantine (12, 22). For NCT02460666, participants continued their current but ineffective antidepressant or psychotherapy treatment and were randomized to receive either Tai chi or health education (23). Treatment duration was 24 weeks for both trials. Participants completed a battery of self-reported and cognitive measures (see Supplementary Table S2) pre- and post-treatment. The primary measure of depression remission in both studies was a HAMD score of 6 or less by end of treatment. The distribution of sociodemographic and illness characteristics did not differ significantly between the two studies (Supplementary Table S1). Most patients in NCT02460666 were maintained on a selective serotonin reuptake inhibitor (SSRI, 20/35, 57.1%), while the remainder received a serotonin norepinephrine reuptake inhibitor (SNRI, 7/35, 20%), norepinephrine and dopamine reuptake inhibitor (NDRI, 2/35, 5.7%), or other treatment (8/35, 22.9%). A total of 28/67 (42%) participants in the combined sample achieved remission of depression by the end of treatment (NCT01902004: 56%; NCT02460666: 29%).

### Image Acquisition

A high-resolution T1-weighted structural brain scan was collected at baseline for each participant using the MPRAGE sequence (3D multi-echo magnetization-prepared rapid gradient-echo sequence). Scans were acquired using Siemens 3T Trio or Prisma systems (Siemens, Erlangen, Germany) with a 32-channel head coil (HEA, HEP) at the Ahmanson and Lovelace Brain Mapping Center at UCLA. Prisma settings: 0.8 mm^3^ isotropic voxel size, TR = 2,500 ms, TE = 1.81:1.79:7.18 ms; FoV = 256 mm; 256 × 256 matrix; TI 1,000 ms; flip angle = 8°. Trio settings: 1 mm^3^ isotropic voxel size, TR = 2,150 ms, TE = 1.74 ms, 3.6, 5.46, and 7.32 ms; FoV = 256 mm; 256 × 256 matrix; TI 1,260 ms; flip angle = 7°. Acquisition time was 8.22 min for Prisma and 5.18 min for Trio scans.

### Image Preprocessing

Freesurfer (version 6.0) (http://surfer.nmr.mgh.harvard.edu) was used for reconstruction of gray matter volumetric measurements at both sites (24). The data cleaning pipeline included the correction of magnetic field in homogeneities, removal of non-brain tissues, segmentation of gray matter from white matter and cerebrospinal fluid, and parcellation of cortical regions using the Desikan–Killany atlas. The reconstructed scans were then carefully inspected for tissue misclassifications and manually corrected as needed. A simple least-square linear regression between raw volumes and the estimated total intracranial volume (eTIV) generated adjusted volumes, a method shown to greatly reduce sex-based volume differences (25).

### Feature Sets

In total, there were seven socio-demographic features, nine medical and mental health illness features, 18 baseline self-reported measures, six cognitive test, and 68 GMV features available in the training and external validation datasets (see Supplementary Table S2). Three feature sets were created: (1) socio-demographic, medical and mental health illness features, and baseline self-reported measures and cognitive tests (designated the “clinical” feature set), (2) GMV features, and (3) combination of all available features.

### Classification Analysis

All analyses were performed in Python (v. 3.8) using the *scikit-learn* (v. 0.23.2) and *mlxtend* packages at default settings (26, 27). Three popular classifiers were selected for comparison with the three feature sets: (1) Support Vector Machine Classifier—Radial Bias Function Kernel (SVMRBF), (2) Random Forest (RF), and (3) L2-regularized Logistic Regression (LR). These algorithms have demonstrated high performance on small datasets in the literature (17, 28). A repeated 5-fold (i.e., 5-folds, 5-repeats) cross-validation approach was used to train and evaluate the classifiers. During splitting, folds were stratified to preserve the proportion of subjects in each target class (e.g., remitter, non-remitter). Data pre-processing steps occurred on the training and test folds independently to avoid against data leakage. Features were filtered to remove those with an absolute intercorrelation of 0.9 (with the features with lesser correlation with predicted target retained) or low variance. Given the excess of features to observations, a wrapper feature selection method was employed. The Boruta algorithm determines relevant features by comparing their predictive performance in a random forest classifier to copies permutated with noise (shadow) (29). Features are ranked and those falling below the maximum importance score of the shadow features or a designated threshold are removed. For the current study, the top 20 features as ranked by the Boruta algorithm were retained for each feature set. Categorical variables were one-hot encoded with 24 missing values imputed by the median value of all other observations. Continuous features were scaled according to the individual feature's quantile range (enables robustness to outliers) and non-normally distributed features were transformed by quantile transformation.

Model performances were estimated by the Area under the ROC Curve (AUC) and Matthews correlation coefficient (MCC) (30). MCC is a more reliable metric than accuracy in binary classification problems as the MCC score is high only if the prediction yields good results in all of the four confusion matrix categories (true positives, false negatives, true negatives, and false positives), proportional to the size of positive elements and the size of negative elements in the dataset (30). Scores were averaged across all folds to determine training and testing performance. The classifiers were refit on the entire training data to calculate final AUC scores and visualized by receiver operator curve.

### Classifier Comparison, Significance Testing, and Feature Information

The significance of the cross-validated performance scores was assessed by permutation analysis. Briefly, predicted targets were permutated 1,000 times to generate a randomized dataset. The percentage of permutations for which the AUC obtained on the randomized data is greater than that obtained using the true data yields the *p*-value. A low *p*-value signifies low likelihood that the model predictions are obtained by chance. Cochran's *Q*-test was performed to determine if the three classifiers differed significantly from each other in performance, followed by *post-hoc* McNemar's Chi-Square test with Bonferroni correction. For all tests, *p* < 0.01 determined significance. The impact of features to model output was explored by calculating Shapley values *via* the *SHAP* package (v. 0.39.0) and visualized by beeswarm plot (31).

## Results

**Figure 1:**
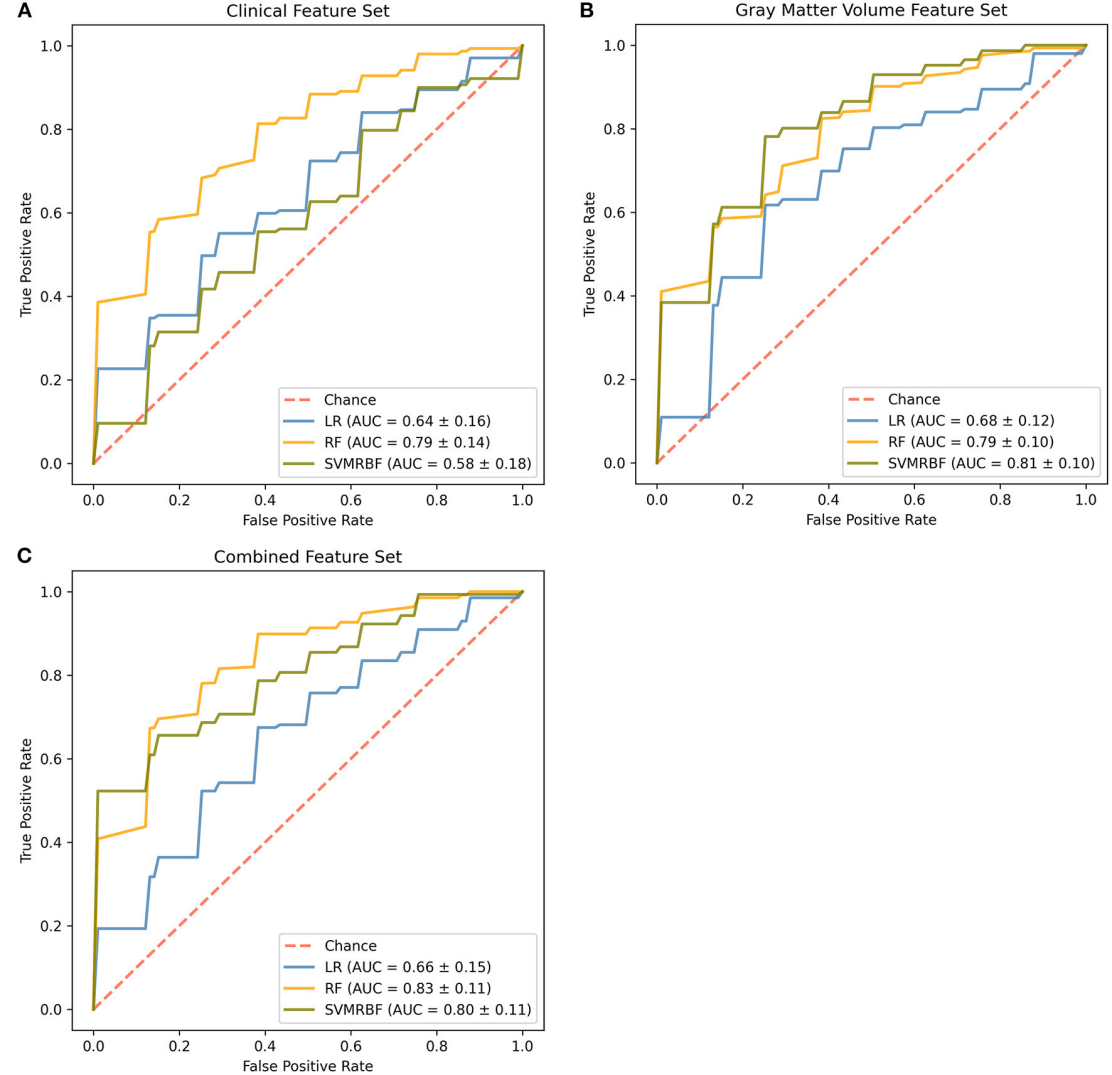
Comparison of classifiers by algorithm and feature set. Evaluated feature sets included **(A)** sociodemographic and clinical features only, **(B)** gray matter volumes only, or **(C)** a combination of all available features. Features were ranked by feature importance and the top 20 from each feature set used for classifier training. The final mean cross-validated area under the cover (AUC) scores are shown for the Logistic Regression (LR, blue), Random Forest (RF, gold), and Support Vector Machine-Radial Bias Function (SVMRBF, olive) classifiers.

The receiver operator curves and final cross-validated AUC scores for each classifier and feature set combination are shown in Figure 1 and Supplementary Table S3. On the clinical feature set, the classifiers performed as follows: LR (Train: AUC 0.84 ± 0.04; Test: AUC 0.65 ± 0.16, MCC 0.19 ± 0.30; Overall: AUC 0.64 ± 0.16); RF (Train: AUC 0.99 ± 0.01; Test: AUC 0.79 ± 0.14, MCC 0.41 ± 0.22; Overall: 0.79 ± 0.14) and SVMRBF (Train: AUC 0.99 ± 0.01; Test: 0.64 ± 0.16, MCC 0.13 ± 0.22; Overall: 0.58 ± 0.18). On the GMV feature set, the classifiers performed as follows: LR (Train: AUC 0.81 ± 0.03; Test: AUC 0.68 ± 0.12, MCC 0.32 ± 0.22; Overall: 0.68 ± 0.12); RF (Train: AUC 0.99 ± 0.01; Test: AUC 0.79 ± 0.10, MCC 0.38 ± 0.24; Overall: 0.79 ± 0.10); and SVMRBF (Train: AUC 0.98 ± 0.01; Test: 0.81 ± 0.10, MCC 0.45 ± 0.20; Overall: 0.81 ± 0.10). On the combined feature set, the classifiers performed as follows: LR (Train: AUC 0.92 ± 0.03; Test: AUC 0.66 ± 0.15, MCC 0.27 ± 0.33; Overall: 0.66 ± 0.15); RF (Train: AUC 0.99 ± 0.00; Test: AUC 0.84 ± 0.11, MCC 0.47 ± 0.29; Overall: 0.83 ± 0.11); and SVMRBF (Train: AUC 0.99 ± 0.00; Test: 0.81 ± 0.11, MCC 0.52 ± 0.22; Overall: 0.80 ± 0.11).

**Figure 2:**
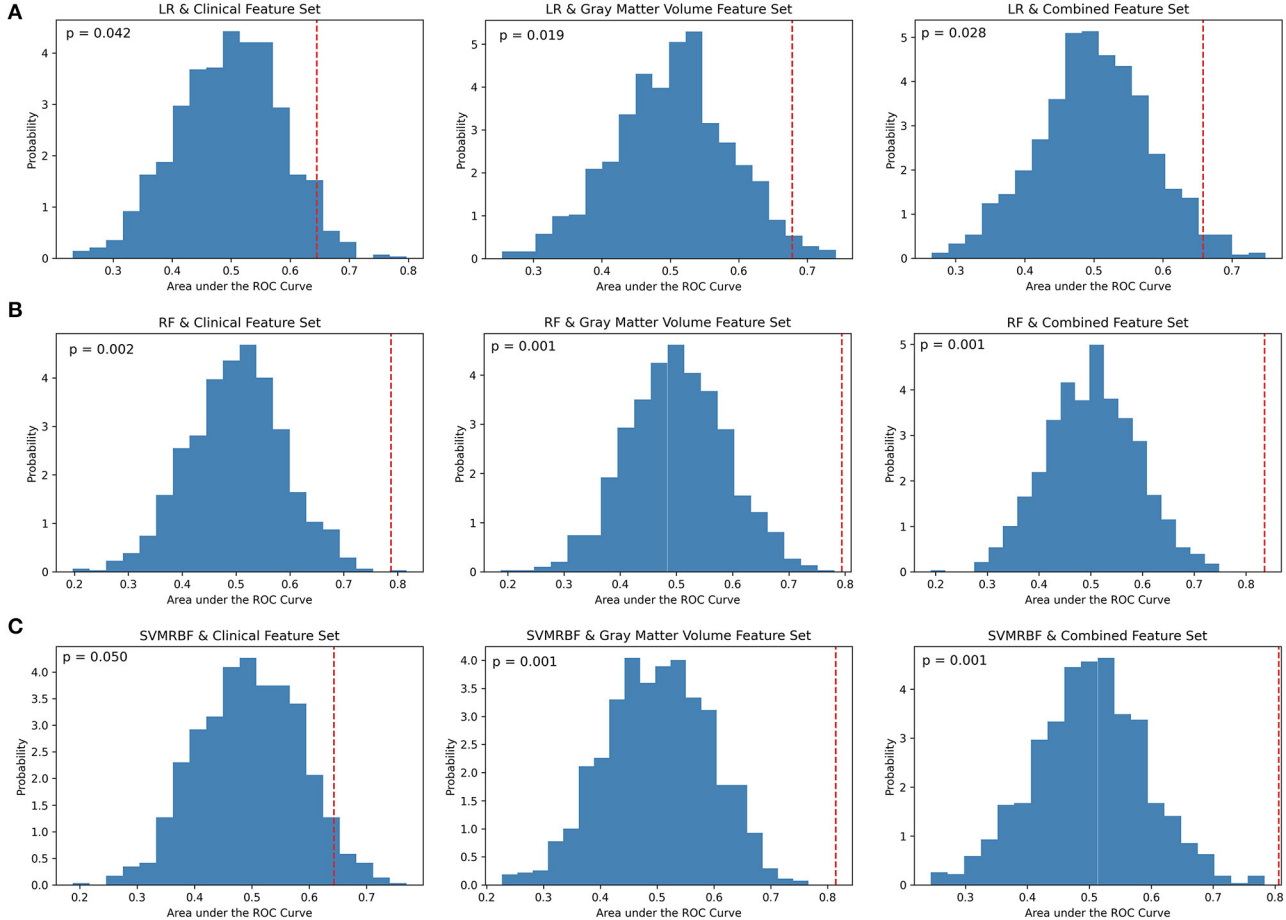
Permutation analysis of cross-validation scores by classifier and feature set. **(A)** Logistic Regression (LR), **(B)** Random Forest (RF), and **(C)** Support Vector Machine-Radial Bias Function (SVMRBF) performance on 1,000 permuted datasets vs. true data was used to calculate the percentage of AUC scores occurring by chance (*p*-value). A *p* < 0.01 determined significance. The dashed red line denotes the mean of AUC scores on the true data compared to the probability distribution of AUC scores calculated on the permuted data.

At a *p* < 0.01 for significance, permutation analysis (Figure 2) indicates that the LR classifier did not achieve performance above chance for any feature set (Clinical: *p* = 0.042; GMV: *p* = 0.019; Combined: *p* = 0.028), the RF classifier achieved significance for all feature subsets (Clinical: *p* = 0.002; GMV: *p* = 0.001; Combined: *p* = 0.001), and the SVMRBF classifier was significant for the GMV and combined feature sets (Clinical: *p* = 0.050; GMV: *p* = 0.001; Combined: *p* = 0.001). Comparison across classifiers using Cochran's test found significance differences for the clinical (*Q*: 18.9, *p* < 0.01), GMV (*Q*: 13.1, *p* < 0.01), and combined feature sets (*Q*: 16.1, *p* < 0.01). For the clinical feature set, *post-hoc* McNemar's Chi-Squared testing found that LR vs. SVMRBF and RF vs. SVMRBF did not differ significantly (Chi2: 3.4, *p* = 0.07; Chi2: 6.1, *p* = 0.01, respectively), but LR vs. RF differed (Chi2: 13.5, *p* < 0.01). For the GMV feature set, LR vs. RF differed significantly (Chi2: 7.6, *p* < 0.01), but not LR vs. SVM RBF (Chi2: 5.8, *p* = 0.02) or RF vs. SVMRBF (Chi2: 0.12, *p* = 0.72). Finally, for the combined feature set, LR vs. SVMRBF and LR vs. RF differed significantly (Chi2: 7.7, *p* < 0.01; Chi2: 7.7, *p* < 0.01), but not RF vs. SVM (Chi2: 0.25; *p* = 0.62).

**Figure 3:**
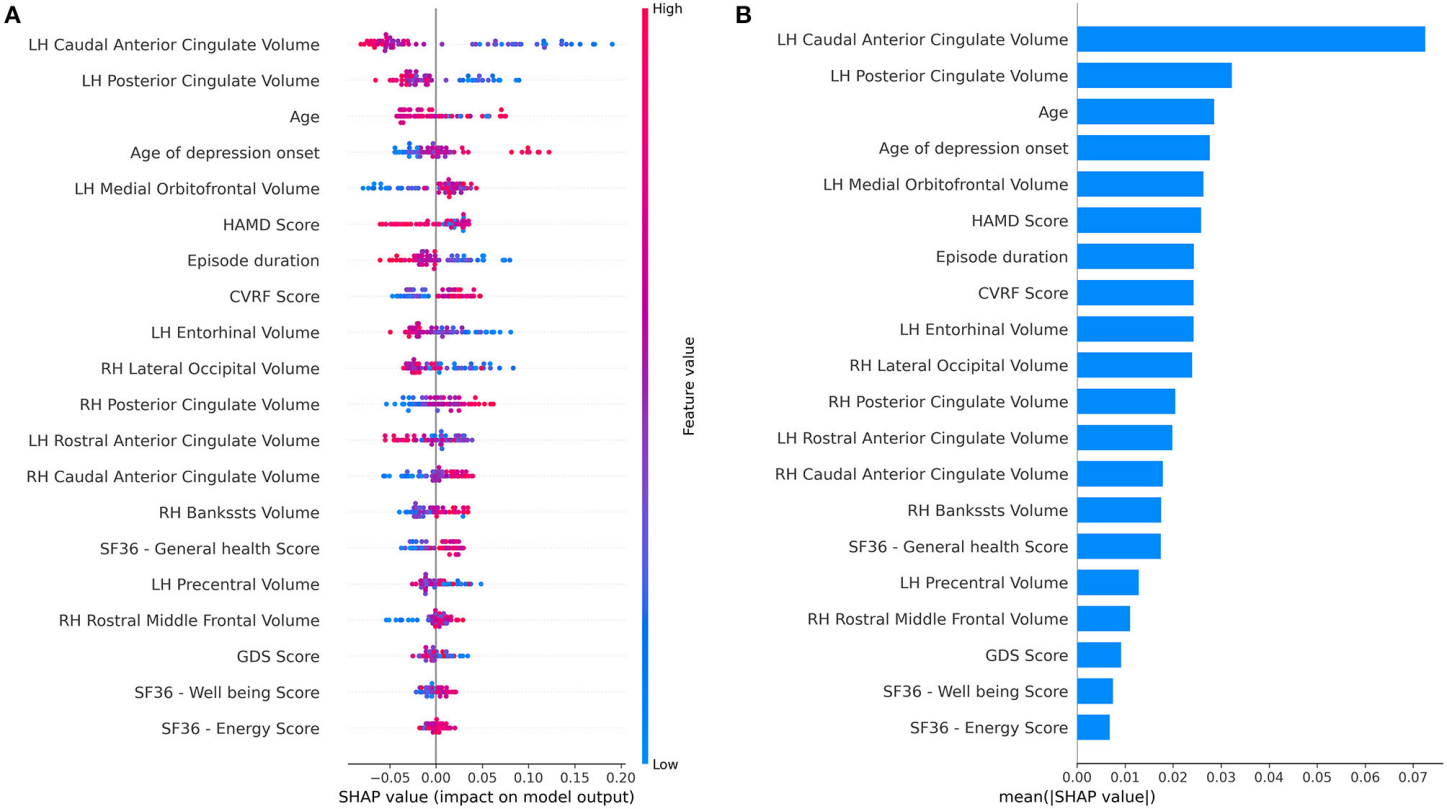
Feature importance summary of random forest classifier using the combined feature set. **(A)** SHAP (SHapley Additive exPlanation) values are ordered by value of a feature to the predictions made by the classifier. The position on the x-axis on shows whether the effect of that value is associated with a higher or lower prediction for a given observation. Red color indicates the feature is high for that observation or low (blue). **(B)** Summary of mean SHAP values or overall magnitude of a feature's impact on prediction of depressive remission.

SHAP (SHapley Additive exPlanation) values were calculated for the RF classifier with the combined feature set (Figure 3). SHAP values reflect the magnitude of a feature's influence on model predictions, not a decrease in model performance as with permutation-based feature performance measures. The most influential feature on prediction of depressive remission was the left-hand caudal anterior cingulate volume, which changes the predicted absolute depression remission probability, on average, by 7%. Other high-ranking features included current age, age of depression onset, baseline HAMD score, current episode duration, and cardiovascular risk factor score, all of which altered remission probability by 2–4%. SHAP values do not permit inference of causality, only correlation with the predicted target.

## Discussion

LLD, like other mood disorders, involves a complicated, multi-directional interplay between biology, psychological, environmental, and social mediators. Considerable heterogeneity exists in clinical phenotypes among patients with LLD, reflective of differing psychobiological pathways to illness. Here, we have demonstrated prediction of treatment response in LLD is improved using a combination of feature types. Our results mirror that of Patel et al. (17), where the authors integrated clinical, cognitive, and MRI data toward improved prediction of diagnosis and treatment response to a 12-week open trial of several different antidepressants in LLD.

The features identified as influencing classifier prediction in the current study corroborate several prior findings in the literature. Age of depression onset and cardiovascular health are among the most notable. LLD encompasses both individuals with early-onset depression (EOD), who develop depressive symptoms before the age of 25 and experience recurrent episodes across lifetime, and individuals with first presentation after age 50–65, or late-onset depression (LOD). The LOD phenotype displays less heritability and a stronger association with underlying cerebrovascular disease with a clinical profile of fronto-subcortical dysfunction, apathy, higher likelihood of progression to dementia, and increased antidepressant resistance (32–34).

Self-reported health-related quality of life (HRQOL) measures (SF36—energy, SF36—emotional well-being) as well as baseline depression severity and chronicity also emerged as informative to prediction, consistent with prior investigations (35–38). Chronic physical disability associates to poor prognosis (39–41). Among the GMVs identified, dysfunction and differences in the anterior cingulate in LLD is well-established (42–44). Entorhinal volume also associates to multiple aspects of LLD, including somatic symptoms and cognitive impairment/conversion to dementia (45–47). Volume of the entorhinal cortex is inversely associated with the number of years since the first episode of depression and associates with treatment-resistant depression in females (6, 45).

The type of response predicted in the current study is general rather than treatment-specific. While the character of the two clinical trial cohorts did not differ substantially in demographics or illness features, the treatment modalities and conditions varied with one group initiating a new SSRI while the other continued their existing antidepressant or therapy and received a new add-on health intervention. Differential treatment response prediction is the goal of the precision medicine approach. However, just as there are converging and diverging pathways to depression, converging and diverging pathways in treatment response (and resistance) are anticipated. Certain data types may offer differing levels of discriminatory predictive power. For example, in a recent study in a sample of 81,630 adults, treatment-specific predictive models from electronic health record data did not perform better than general treatment response models (48). A classifier capable of predicting treatment response to a focused range of options (e.g., SSRIs) could arguably hold higher clinical utility in practice than one that predicts response to a single agent (49, 50).

The current work has several strengths, including the rigor of the analysis. Machine learning algorithms possess known variability in their tolerance for number of features, multi-collinearity, and noise. The RF classifier, for example, performed well-across all feature sets and demonstrated the least degradation in performance (generalization error) on the testing data. The primary limitations of the study are the small sample size and lack of a dataset with similar features for external validation. Cross-validation is only an estimate of performance on unseen data. The generalizability of a model cannot be fully determined without validation in an external dataset (51). Additionally, “small” data is prone to overfitting, even with robust feature selection and cross-validation. For the current work, a static number of features were employed in each feature set to permit comparison across classifiers. In moving from exploratory analysis to development of an optimized model, features could be even more aggressively reduced, hyperparameters tuned (e.g., limiting the maximum depth of the branching of the RF classifier, the number of support vectors for SVMRBF), and models combined (ensemble modeling) to further reduce overfitting.

## Conclusion

The current preliminary study into the use of ML to identify predictors of treatment response in late-life depression indicates that integration of clinical and structural MRI significantly increases predictive capability. Timely treatment selection in LLD is critical to preservation of quality of life and cognitive capacity. The current results suggest machine learning coupled with multi-modal data are a promising avenue for the development of a non-invasive, precision approach to illness management.

## Data Availability Statement

The data analyzed in this study is subject to the following licenses/restrictions: Protected health information, clinical trial data. Requests to access these datasets should be directed to Helen Lavretsky (hlavretsky@mednet.ucla.edu).

## Author Contributions

HL and KN conceived of the study. AG completed the analyses. AP and BK-S assisted with data processing. All authors contributed to the writing of the manuscript and interpretation of results.

## Conflict of Interest

The authors declare that the research was conducted in the absence of any commercial or financial relationships that could be construed as a potential conflict of interest.

## Publisher's Note

All claims expressed in this article are solely those of the authors and do not necessarily represent those of their affiliated organizations, or those of the publisher, the editors and the reviewers. Any product that may be evaluated in this article, or claim that may be made by its manufacturer, is not guaranteed or endorsed by the publisher.
