# Supplementary material for: Machine Learning Prediction of Treatment Outcome in Late-Life Depression
Source: Front Psychiatry. 2021 Oct 20;12:738494. doi: 10.3389/fpsyt.2021.738494 (PMC8563624; doi:10.3389/fpsyt.2021.738494)
Supplement: Supplementary file 1 [file Table_1.pdf]

Supplemental Table S1. Sociodemographic, baseline depression severity, and remission rates of sample

Characteristic	Overall, N = 67[1]	NCT01902004, N = 32	NCT02460666, N = 35	p-val[2]
Age, years	69.85 (6.92)	71.16 (7.28)	68.66 (6.45)	0.095
Sex				0.036
Female	46 / 67 (69%)	18 / 32 (56%)	28 / 35 (80%)	
Male	21 / 67 (31%)	14 / 32 (44%)	7 / 35 (20%)	
Race				0.2
White/Caucasian	53 / 67 (79%)	25 / 32 (78%)	28 / 35 (80%)	
Hispanic	7 / 67 (10%)	5 / 32 (16%)	2 / 35 (5.7%)	
Asian/Pacific Islander	3 / 67 (4.5%)	0 / 32 (0%)	3 / 35 (8.6%)	
Black/African American	3 / 67 (4.5%)	1 / 32 (3.1%)	2 / 35 (5.7%)	
Other	1 / 67 (1.5%)	1 / 32 (3.1%)	0 / 35 (0%)	
Education, years	15.94 (2.11)	15.75 (2.16)	16.11 (2.08)	0.5
Marital status				0.8
Married	35 / 67 (52%)	16 / 32 (50%)	19 / 35 (54%)	
Divorced	20 / 67 (30%)	9 / 32 (28%)	11 / 35 (31%)	
Never been married	11 / 67 (16%)	6 / 32 (19%)	5 / 35 (14%)	
Widowed	1 / 67 (1.5%)	1 / 32 (3.1%)	0 / 35 (0%)	
HAMD, baseline	18.06 (3.17)	17.59 (2.37)	18.49 (3.74)	>0.9
HAMD, remission (≤6) at 24w	28 / 67 (42%)	18 / 32 (56%)	10 / 35 (29%)	0.022

[1]Mean (SD); n / N (%)

[2]Wilcoxon rank sum test (continuous); Pearson's Chi-squared test or Fisher's exact test (categorical)

Abbreviations: HAMD: Hamilton Depression Rating Scale
