# Supplementary material for: Machine Learning Prediction of Treatment Outcome in Late-Life Depression
Source: Front Psychiatry. 2021 Oct 20;12:738494. doi: 10.3389/fpsyt.2021.738494 (PMC8563624; doi:10.3389/fpsyt.2021.738494)
Supplement: Supplementary file 2 [file Table_2.pdf]

Supplemental Table S2. Features available in the training dataset

Features	Type
Sociodemographic	Age, sex, race, handedness, education, marital status, living status
Medical	Hypertension, Alcohol use
Depression	Family history of depression, current stressors, current bereavement, current episode duration, chronicity of current episode, age of depression onset, prior episodes of depression
Self-reported measures	AES: Apathy Evaluation Scale; HAM-A: Hamilton Anxiety Rating Scale; CD-RISC: Connor-Davidson Resilience Scale; CIRS-G: Cumulative Illness Rating Scale for Geriatrics; CVRF: Cerebrovascular Risk; SF-36: 36-Item Short Form Survey (separated by domain); GDS: Geriatric Depression Scale; AES: Apathy Evaluation Scale; CGI: Clinical Global Impressions; HAMD: Hamilton Depression Rating Scale; MMSE: Mini Mental Status Exam
Cognitive tests	Learning, delayed recall, language, processing speed, executive functioning, visual spatial
Gray matter volumes (right & left) for the following regions:	Transverse temporal, temporal pole, supramarginal, superiortemporal, superiorparietal, superiorfrontal, rostralmiddlefrontal, rostralanteriorcingulate, precuneus, precentral, posteriorcingulate, postcentral, pericalcarine, parstriangularis, parsorbitalis, parsopercularis, parahippocampal, paracentral, middletemporal, medialorbitofrontal, lingual, lateralorbitofrontal, lateraloccipital, isthmuscingulate, insula, inferiortemporal, inferiorparietal, fusiform, frontalpole, entorhinal, cuneus, caudalmiddlefrontal, caudalanteriorcingulate, bankssts
