# Supplementary material for: Machine Learning Prediction of Treatment Outcome in Late-Life Depression
Source: Front Psychiatry. 2021 Oct 20;12:738494. doi: 10.3389/fpsyt.2021.738494 (PMC8563624; doi:10.3389/fpsyt.2021.738494)
Supplement: Supplementary file 3 [file Table_3.pdf]

Supplemental Table S3. Classifier cross-validation training and testing results by feature set

Feature Set	Classifier	Train AUC[1]	Test AUC[1]	Test MCC[1]
Clinical	LR	0.84 (0.04)	0.65 (0.16)	0.19 (0.30)
	RF	0.99 (0.01)	0.79 (0.14)	0.41 (0.22)
	SVMRBF	0.99 (0.01)	0.64 (0.14)	0.13 (0.22)
Gray matter volume	LR	0.81 (0.03)	0.68 (0.12)	0.32 (0.22)
	RF	0.99 (0.01)	0.79 (0.10)	0.38 (0.24)
	SVMRBF	0.98 (0.01)	0.81 (0.10)	0.45 (0.20)
Combined	LR	0.92 (0.03)	0.66 (0.15)	0.27 (0.33)
	RF	0.99 (0.01)	0.84 (0.11)	0.47 (0.29)
	SVMRBF	0.99 (0.01)	0.81 (0.11)	0.52 (0.22)

[1] Mean (SD) over 5-fold repeated cross-validation
Abbreviations: LR: Logistic Regression; RF: Random Forest; SVMRBF: Support Vector Machine-Radial Bias Function. AUC: Area under the ROC curve; MCC: Matthews Correlation Coefficient
